# Use of Osteopathic Manipulation for Treatment of Chronic Shoulder Injury Related to Vaccine Administration

**DOI:** 10.7759/cureus.9156

**Published:** 2020-07-12

**Authors:** Simrat Veera, Justin Chin, Lina Kleyn, Salvatore Spinelli, Leonid Tafler

**Affiliations:** 1 Pediatrics, Goryeb Children’s Hospital - Atlantic Health System, Morristown, USA; 2 Family Medicine, LifeLong Medical Care, Richmond, USA; 3 Medical Education, Lake Erie College of Osteopathic Medicine, Erie, USA; 4 Family Medicine, Morristown Medical Center - Atlantic Health System, Morristown, USA; 5 Primary Care, Touro College of Osteopathic Medicine, New York, USA

**Keywords:** sirva, omm, spencer technique, shoulder, vaccination, immunization, anesthesia, shoulder dysfunction, range of motion, pain

## Abstract

Shoulder injury related to vaccine administration (SIRVA) is an increasingly reported phenomenon that causes inflammation of surrounding structures, along with pain and decreased range of motion of the affected shoulder. Current literature emphasizes proper injection techniques and locations to decrease incidence; however, there is limited information available on successful treatments. The aim of this report is to describe a case of SIRVA and review treatment options, specifically the role of osteopathic manipulative medicine (OMM) in the resolution of symptoms refractory to standard care. Here we present a case of chronic SIRVA in a 58-year-old female due to a poorly administered influenza vaccination with emphasis on a stepwise osteopathic therapy approach as a lasting treatment to decrease the effects of the inflammatory process and improve daily function of the shoulder. OMM, with the option of anesthesia, can be performed in outpatient family medicine practices as a noninvasive and safe adjunct treatment. Specifically, the Spencer technique has been shown to improve shoulder-related pathologies that include adhesions, capsulitis, and inflammation and was used in this case under anesthesia. The patient reported good improvement in her symptoms and increased range of motion. SIRVA is an underdiagnosed phenomenon that involves inflammation of surrounding structures after a vaccine administration. In chronic cases, such as in this patient, OMM may be enhanced with the use of anesthesia to optimize the treatment’s effect on scar tissue and fibrosis.

## Introduction

Modern vaccinations are commonly administered in the deltoid muscle, using landmarking to find the optimal location [1]. Common post-administration reactions to intramuscular vaccine injections can include pain, erythema, and swelling, all of which tend to resolve within 24-48 hours [2]. Shoulder injury related to vaccine administration (SIRVA) is a rarer complication that occurs within 48 hours after injection and presents with long-term shoulder pain and restricted range of motion (ROM) [3,4]. Caused by improper landmarking and/or intramuscular injection techniques, SIRVA can result in post-injection complications, such as shoulder bursitis, adhesive capsulitis and inflammation to adjacent structures [5]. Current literature has emphasized prevention of these adverse events through proper injection techniques, with limited research done on treatments targeted towards SIRVA sequelae [6,7]. The conventional treatment for SIRVA primarily focuses on a stepwise approach through the use of anti-inflammatory medications, physical therapy, and/or surgical intervention [8]. Greater than 50% of patients, however, may report residual symptoms despite these interventions [9].

Osteopathic manipulative medicine (OMM) describes the field of medicine that utilizes musculoskeletal adjustment techniques to restore health and functionality to the body [10]. Osteopathic assessments include a thorough examination of neuromuscular and skeletal pathologies, with engagement of fascial and connective tissues [11,12]. Traditionally done awake, anesthesia can be introduced to facilitate muscle relaxation and aid in patient comfort. OMM with anesthesia allows for physicians to breakup scarred tissue built up through chronic inflammatory processes, thus improving overall movement [13]. The use and efficacy of OMM in a stepwise fashion has not been well reported in the treatment of SIRVA. Here, we describe a case of chronic SIRVA after administration of an influenza vaccine, resulting in severe pain and decreased ROM, both of which were resolved using OMM with anesthesia.

## Case presentation

**Figure 1:**
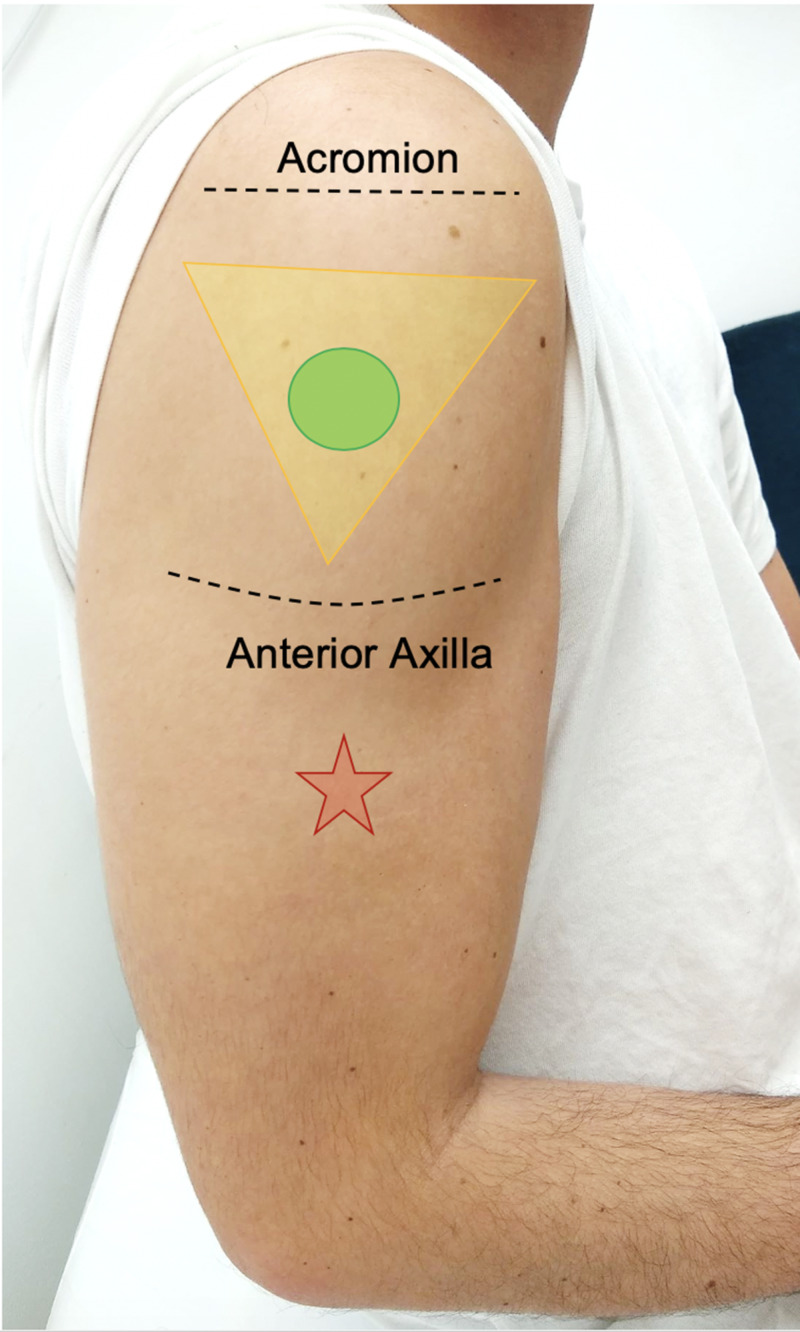
Appropriate location for vaccine administration in the deltoid, using the acromion as a landmark. The base of the triangle is approximately two to three fingers below the acromion (top line) to avoid the shoulder capsule and bursa. The apex of the triangle lines up with the anterior axilla (bottom line) to form a triangle (yellow) and ideal injection site (green circle) atop the deltoid muscle for injection. The patient pointed to an injection site (red star) that was a subjective two inches below the deltoid muscle.

A 58-year-old female with a past medical history of hypertension presented with a four-month history of right shoulder pain, complicated with paresthesia to the right hand and decreased shoulder ROM. Her symptoms had impacted her ability to perform daily activities, including her duties as a nurse. The symptoms started within 24 hours of receiving an inactivated influenza vaccine, subjectively placed two inches below her deltoid (Figure 1). Despite numerous visits to the emergency room, her primary care physician, and an orthopedic surgeon, her attribution of the pain to the vaccine injection was not taken into consideration. Before seeing an osteopathic physician, she was ultimately diagnosed with a cervical spine strain and right shoulder tendonitis. In that time, she had been treated with corticosteroid injections in the acromial bursa, nonsteroidal anti-inflammatory drugs (NSAIDs) in varying strengths, ongoing physical therapy, and was advised to schedule surgical intervention due to minimal improvement. At that time, the patient sought out an osteopathic family practice for a reassessment.

Surgical, family, and social history were noncontributory. Osteopathic physical exam revealed no visible skin changes or swelling. There was limited pan-directional passive and active ROM in the right shoulder, with marked limitation on abduction and pain on palpation of the injected site. ROM at the right elbow joint was unaffected. Light and deep pressures were felt equally along the right arm and hand. Spurling compression test for cervical radiculopathy was negative.

**Figure 2:**
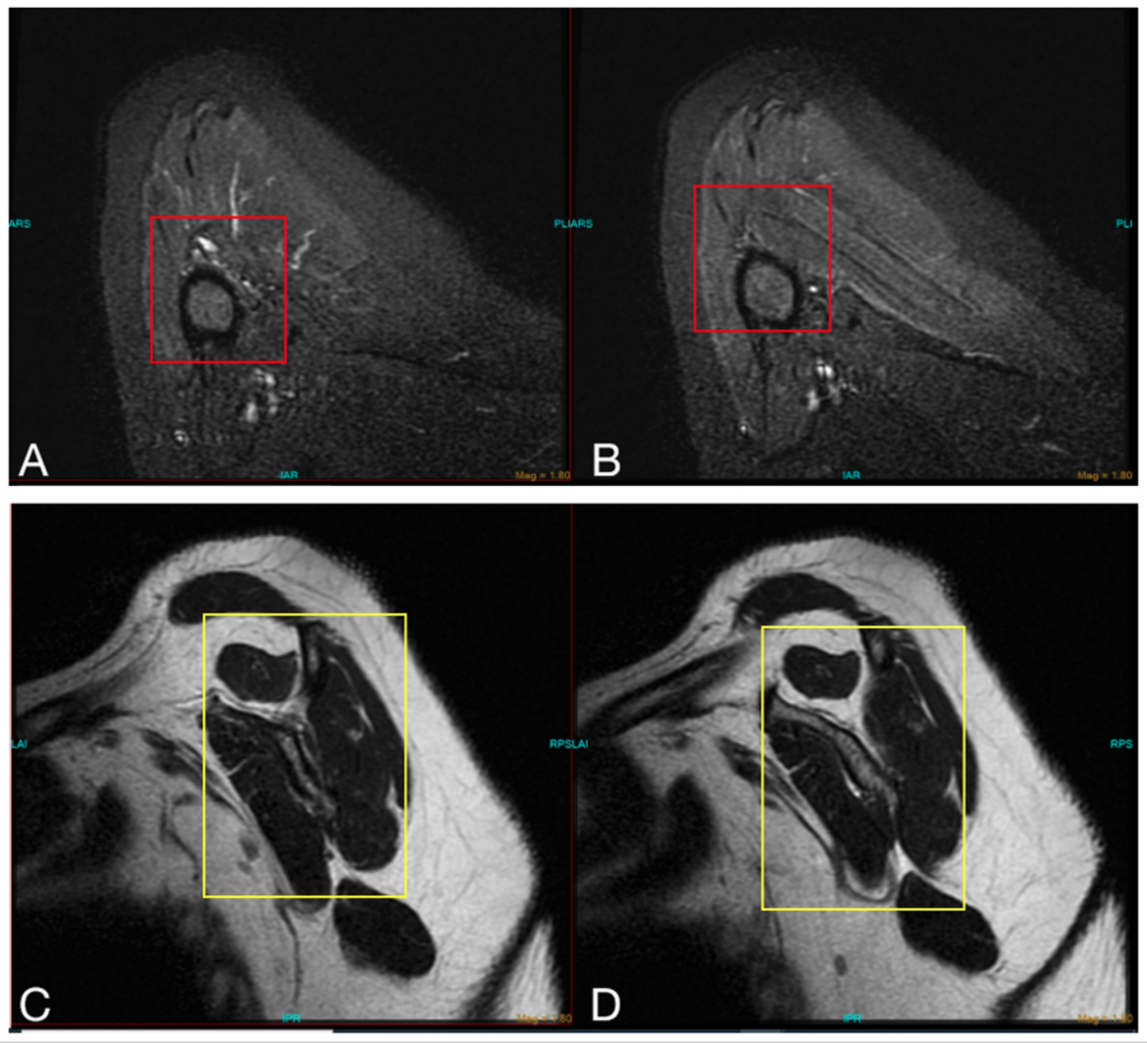
MRI of the right shoulder with multiple views demonstrating (A and B) mild rotator cuff tendinopathy with a partial thickness rotator cuff tear (red box) and (C and D) inflammation of the biceps muscle (yellow box).

In reviewing her radiological history, plain radiographs of the cervical spine were unremarkable. Subsequent MRI of the right shoulder, six weeks after the vaccine was administered, demonstrated mild rotator cuff tendinopathy with a partial thickness rotator cuff tear, moderate bicipital tenosynovitis, and glenohumeral joint effusion. No fracture or dislocations were appreciated (Figure 2).

**Figure 3:**
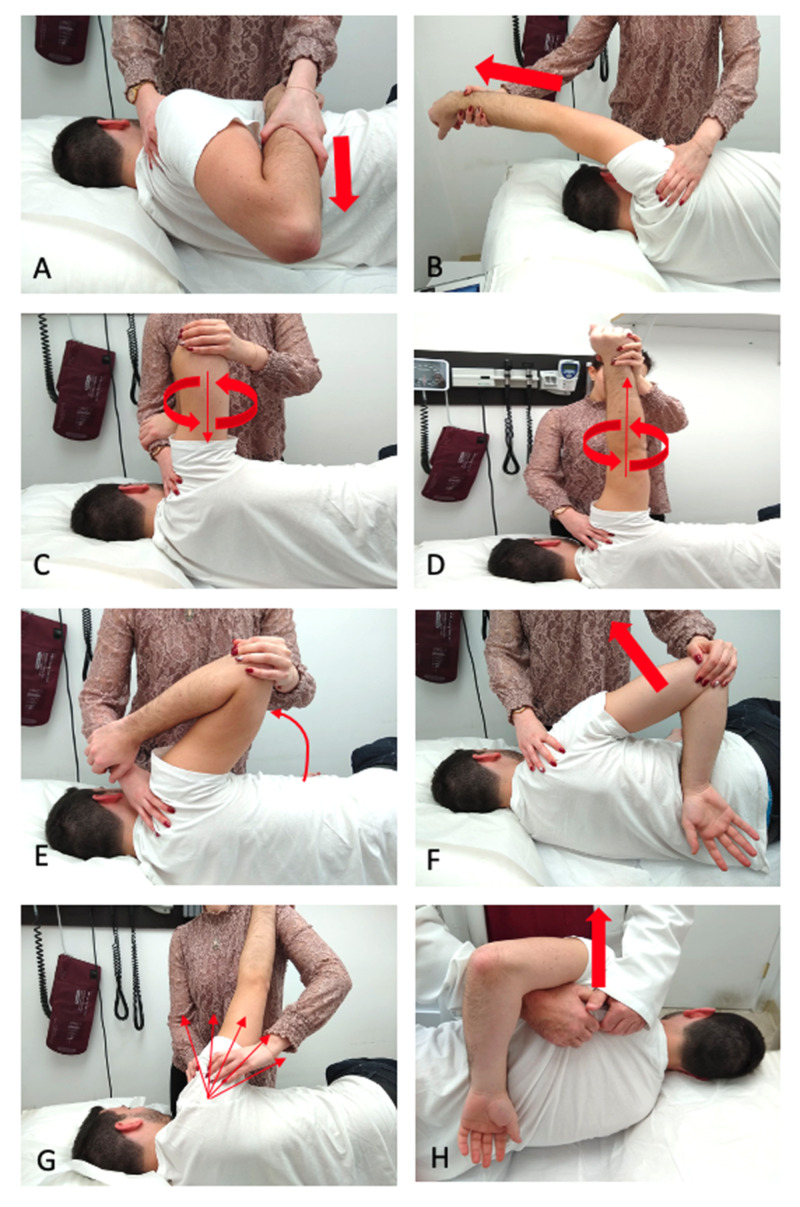
Demonstration of the Spencer technique used to increase range of motion of the glenohumeral joint. Articulations are performed in seven consecutive stages: (A) extension, (B) flexion, (C) circumduction with compression, (D) circumduction with traction, (E) abduction, (F) internal rotation, (G) lymphatic pump. Each stage is proceeded by active resistance in that motion. Additional manipulation, such as (H) scapula lift, may be added to supplement the treatment.

After her initial osteopathic assessment, the patient elected for an injection of 1.0 mL methylprednisolone and 4.0 mL of 2% lidocaine in the area of vaccination to reduce local inflammation at the site of trauma. In subsequent follow-ups, she was prescribed a 4 mg oral methylprednisolone dose pack. When symptoms did not improve, osteopathic manipulation treatment was performed weekly for four months, in which a modified Spencer’s technique sequence with muscle energy was performed (Figure 3). During this time, the patient also continued physical therapy and started acupuncture treatments to the right shoulder. She reported mild alleviation of pain and increased movement in select ranges of motion, but she was still unable to perform daily activities or return to work.

**Figure 4:**
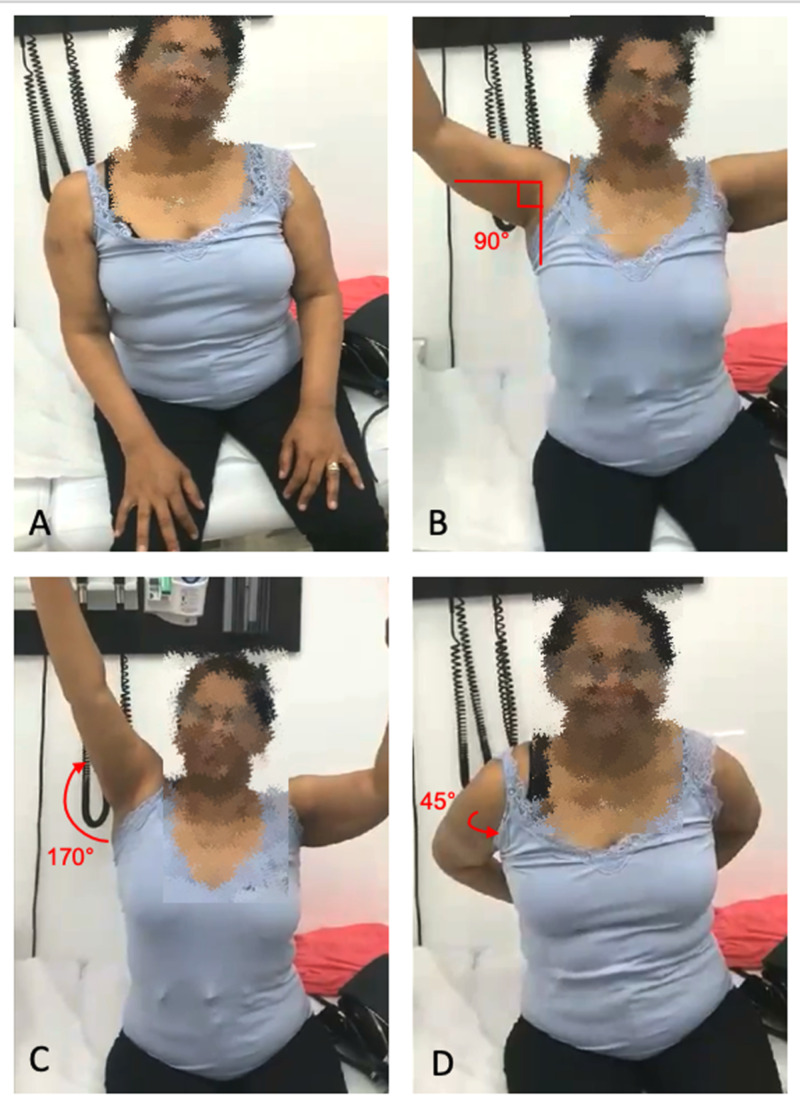
(A) Initial presentation showing right shoulder restricted in motion in all directions, especially abduction. (B) Post osteopathic manipulative medicine (OMM) with anesthesia showing right shoulder active abduction to 90°. (C) Post OMM with anesthesia showing right shoulder active flexion to 170°. (D) Post OMM with anesthesia showing right shoulder active internal rotation to 45°. Patient consent was obtained for images.

After four months of standard OMM techniques, OMM under general anesthesia, in this case propofol, was offered due to minimal improvement. The same modified Spencer technique was performed, along with additional manipulations such as a scapula lift. Status post-anesthesia recovery from the procedure, the patient was assessed for pain and ROM. Compared to the minimal pan-directional movement on initial presentation, the right shoulder joint was able to abduct passively to 180° and actively to 90°, flex passively to 180° and actively to 170°, and internally rotate to 45° in both active and passive ROM (Figure 4). For one month after the procedure, the patient continued weekly acupuncture treatments, nonanesthetic osteopathic manipulations, and physical therapy to the right shoulder, with continued improvement of her symptoms. One additional osteopathic manipulation under anesthesia was performed at the end of the one-month period after which the patient demonstrated near full ROM of the right shoulder with only mild pain after 130° of flexion/abduction. She continues to have occasional OMM and physical therapy sessions; however, her pain and other symptoms have largely resolved. 

Patient consent for this case presentation and pictures were obtained.

## Discussion

According to the Centers for Disease Control, more than 3.1 billion vaccines have been administered between 2006 and 2016 [14]. In the same decade, only 112 cases of SIRVA have been adjudicated by the Office of Special Masters of the US Court of Federal Claims, with half of those cases occurring in the past two years [14]. The true incidence and prevalence in the United States of SIRVA are unclear as research has primarily been reported as isolated case series [15]. SIRVA symptoms mimic multiple neuromusculoskeletal pathologies such as cervical radiculopathy, rotator cuff strain, and adhesive capsulitis, which can lead to underdiagnosis and misdiagnosis [14,15]. Thorough clinical history and elicitation of the inciting event is critical diagnosing SIRVA. Despite limited studies, a chronic inflammatory reaction following injection of antigenic material in synovial tissues has been the prevailing proposed mechanism for SIRVA [8].

Multiple reports and reviews on SIRVA emphasize the need for proper injection techniques to avoid adverse injuries [3]. Common themes include appropriate needle length, using the acromion as a landmark, the motion and angle with which the needle is injected, and standardized skin preparation [4,5]. The patient in this case study subjectively reports an injection site below the recommended area. Standard treatment regimens for SIRVA, typically done in a stepwise escalating fashion, utilize NSAIDs, corticosteroid injections, physical therapy, and surgical intervention [7]. More than 50% of patients nationally, however, continue to complain of residual symptoms despite attempting all or some combination of the aforementioned options [8]. As in our case, the patient had exhausted the standard care recommendations, just short of surgery, thus seeking out an alternative approach to manage SIRVA.

OMM identifies and aims to treat “somatic dysfunctions” or physical responses on the musculoskeletal system due to improper functioning or imbalance in the body [10]. Since its inception in the 1890s, evidence-based modalities have been developed as primary or adjunctive therapies whose aims are to restore function and homeostasis to an affected area and the body as a whole [16]. The Spencer technique is a common nonsurgical treatment to effectively address inflammatory shoulder dysfunctions such as adhesive capsulitis, bursitis, and tenosynovitis [17]. This method aims to lyse fibrotic tissue and adhesions through repeated articulations of the glenohumeral joint in seven stages, with modifications for active resistance, number of repetitions, and targeting surrounding structures added as needed. The case patient’s duration and presentation of symptoms were suggestive of a chronic inflammatory process, in which the Spencer technique has been proven to be beneficial. Due to the patient’s severity and minimal improvement after conventional therapy, the use of anesthesia in conjunction with OMM was a reasonable alternative in lieu of invasive surgery. By introducing anesthesia into the manipulation, the Spencer technique is optimized as patient discomfort and guarding may be decreased.

**Figure 5:**
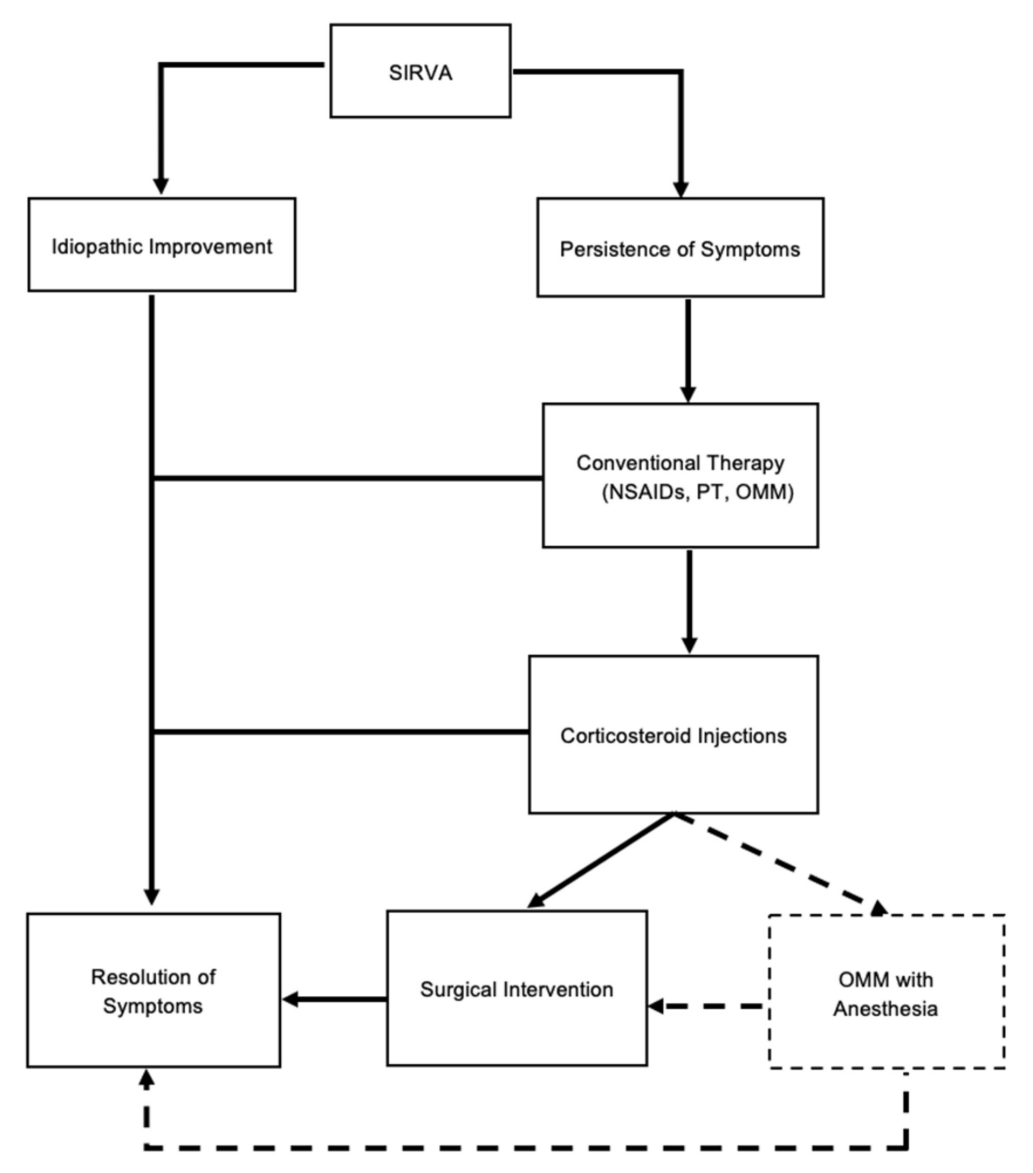
Schematic diagram of stepwise SIRVA treatment incorporating OMM with anesthesia (dotted line). NSAID, nonsteroidal anti-inflammatory drug; OMM, osteopathic manipulative medicine; PT, physical therapy; SIRVA, shoulder injury related to vaccine administration.

The addition of anesthesia with musculoskeletal manipulation has been in practice since the 1930s by orthopedic surgeons, osteopathic physicians, and more recently, chiropractors, as an alternative to failed conservative methods and surgical intervention [18]. Propofol (Diprivan) and midazolam (Versed) are common agents used in the procedure, similar to their administration in short procedures such as colonoscopies [18]. Despite the poorly understood mechanism of action of general anesthetics, the interruption of nerve signals between the brain and body results in a sedative state, and has been disputed to act on the voltage-gated sodium channels in muscle spindles, directly relaxing skeletal muscle [19]. With complete relaxation of the muscle reflexes and pain fibers, larger articulatory motions can be performed to lyse fibrotic and scarred tissue caused by chronic inflammation [20]. In addition, the patient’s pain is theoretically in control by the sedative effect. While this technique was successful in restoring ROM function of the glenohumeral joint and reducing pain levels in our patient, manipulation under anesthesia should be approached as an end alternative after standard managements, including nonmedicated OMM, are attempted (Figure 5). Limited research has been done on medication-assisted OMM, with even less consensus and standardization on its application and practice in a family practice setting. Further studies are needed to assess its standardized application and potential as an adjuvant to osteopathic manipulation.

## Conclusions

Shoulder inflammation related to vaccine administration, SIRVA, is an underdiagnosed phenomenon that involves inflammation of surrounding structures after a vaccine administration. Conventional therapies for SIRVA, such as NSAIDs, corticosteroid injections, and physical therapy, may not be enough to manage symptoms in chronic settings. In these cases, commonly used OMM modalities, such as the Spencer technique, may be a useful procedure to perform in adjunct to stepwise allopathic regimens. In chronic cases, such as in this patient, OMM may be enhanced with the use of anesthesia to optimize the treatment’s effect on scar tissue and fibrosis. If assessed and performed correctly, patients with chronic SIRVA may respond well to this noninvasive alternative to surgical intervention.
